# Separated Siamese Twins: Intronic Small Nucleolar RNAs and Matched Host Genes May be Altered in Conjunction or Separately in Multiple Cancer Types

**DOI:** 10.3390/cells9020387

**Published:** 2020-02-07

**Authors:** Marianna Penzo, Rosanna Clima, Davide Trerè, Lorenzo Montanaro

**Affiliations:** 1 Laboratorio di Patologia Clinica, Dipartimento di Medicina Specialistica, Diagnostica e Sperimentale, Università di Bologna, Via Giuseppe Massarenti 9, 40138 Bologna, Italy; davide.trere@unibo.it (D.T.); lorenzo.montanaro@unibo.it (L.M.); 2 Centro di Ricerca Biomedica Applicata (CRBA), Policlinico Universitario di S. Orsola, Università di Bologna, Via Massarenti 9, 40138 Bologna, Italy; 3 BROWSer S.r.l. –Bioinformatics Resource for Omics Wide Services, c/o Dipartimento di Bioscienze, Biotecnologie e Biofarmaceutica, Università di Bari “Aldo Moro”, Via E. Orabona 4, 70126 Bari, Italy; r.clima@browser-bioinf.com

**Keywords:** cancer, intronic snoRNA, H/ACA box, C/D box, CNA, copy number alteration, amplification, deletion, host gene, expression alteration

## Abstract

Small nucleolar RNAs (snoRNAs) are non-coding RNAs involved in RNA modification and processing. Approximately half of the so far identified snoRNA genes map within the intronic regions of host genes, and their expression, as well as the expression of their host genes, is dependent on transcript splicing and maturation. Growing evidence indicates that mutations and/or deregulations that affect snoRNAs, as well as host genes, play a significant role in oncogenesis. Among the possible factors underlying snoRNA/host gene expression deregulation is copy number alteration (CNA). We analyzed the data available in The Cancer Genome Atlas database, relative to CNA and expression of 295 snoRNA/host gene couples in 10 cancer types, to understand whether the genetic or expression alteration of snoRNAs and their matched host genes would have overlapping trends. Our results show that, counterintuitively, copy number and expression alterations of snoRNAs and matched host genes are not necessarily coupled. In addition, some snoRNA/host genes are mutated and overexpressed recurrently in multiple cancer types. Our findings suggest that the differential contribution to cancer development of both snoRNAs and host genes should always be considered, and that snoRNAs and their host genes may contribute to cancer development in conjunction or independently.

## 1. Introduction

Small nucleolar RNAs (snoRNAs) are non-coding RNAs of 60-300 nucleotides in length which are involved in fundamental molecular processes, such as RNA modification and processing. Even though some snoRNAs have been known for decades, hundreds have only recently been identified, and to date their number is over 700. Approximately half of the so-far identified snoRNA genes map within the intronic regions of both protein-coding genes or long-noncoding sequences whose function is, in some cases, poorly understood. Most snoRNA host genes belong to the 5′-terminal oligopyrimidine (5′-TOP) family, whose transcripts start with an oligopyrimidine tract [1,2], allowing for a specific expression regulation during cellular growth and proliferation [3]. Many of the protein-coding genes encode for ribosomal proteins, translation factors, or other proteins involved in ribosome biogenesis, suggesting the need for a balanced synthesis of these proteins and their corresponding snoRNAs. Intronic snoRNAs are transcribed by RNA polymerase II (Pol II) and are released from their transcripts (mRNAs or long noncoding RNAs (lncRNAs) precursors depending on the type of the host gene) through a process involving splicing and trimming [4,5].

The remaining half of snoRNA genes map within DNA intergenic regions, are endowed with independent promoters and are transcribed by Pol II or Pol III either as independent units, or as polycistronic structures [5].

The vast majority of snoRNAs can be divided into two main structural classes: H/ACA box snoRNAs, characterized by a double hairpin, separated by a hinge region containing the H box, and an ACA box close to the 3′ terminus; and C/D box snoRNAs, containing the C box close to the 5′ terminus, and the D box near to the 3′ end (reviewed in [6]). snoRNAs bind to specific proteins to form small nucleolar ribonucleoproteins (snoRNPs), complexes designated for RNA modification. H/ACA snoRNAs bind to NOP10, GAR1, NHP2, and dyskerin (DKC1), and guide the pseudouridylase activity to modify specific uridines (reviewed in [7]). Similarly, C/D box snoRNAs bind to NOP56, NOP58, 15.5 kDa, and Fibrillarin (FBL), and guide the 2′-*O*-Methylation of specific rRNA sugar residues [8]. In both cases, the target of the modification is determined by sequence complementarity between the guide and the target RNA, allowing Watson–Crick base pairing. However, a subgroup of snoRNAs, called “orphan” snoRNAs, do not match any RNA sequences, and their targets cannot be predicted [9]. Most of the known snoRNA targets are ribosomal RNAs (rRNAs), but recently other target types, such as messenger RNAs (mRNAs), have been identified [10]. In rRNA, 200 residues are modified by pseudouridylation or 2′-*O*-methylation, and these modifications are regarded to as fundamental steps in rRNA maturation and ribosome assembly, and for ribosomal function (reviewed in [6]).

In addition, snoRNAs can be processed further to generate snoRNA-derived RNAs (sdRNAs), with features and functions similar to microRNAs (miRNAs) [11]. It is plausible that this processing is preferentially reserved to orphan snoRNAs, but it cannot be excluded *a priori* for other snoRNAs.

Genetic instability is a common feature in many cancer types; genetic alterations involving protein-coding genes (among which are snoRNA host genes) are, in most cases, very well characterized. Conversely, the contribution to cancer onset of alterations occurring in non-coding DNA regions, is now becoming more and more clear after many years of neglect. Growing evidence indicates that mutations and/or deregulation affecting snoRNAs may play a significant role in oncogenesis, even though the mechanism by which snoRNA deregulation ultimately contributes to cancer onset remains, in most cases, unknown [12]. Deregulation of specific snoRNAs has been proposed to help in generating a cancer-prone setting by affecting different cancer-related cellular processes, like those controlling cellular growth, death, invasion and angiogenesis [13,14,15].

In this sense, many of the studies conducted on this topic have focused on the evaluation of the expression of snoRNAs and their matched host genes [16,17,18,19], while others were limited to the study of the mutations of a single snoRNA in a specific cancer setting [16,20,21,22]. In addition, co-occurrence of genetic alterations in snoRNAs and matched host genes should be taken under consideration, particularly for host genes for which a pro-oncogenic or tumor suppressive function has been ascribed. Here, we analyzed the copy number alterations (CNAs, i.e., amplifications and deep deletions) of 295 matched snoRNA-host gene couplets in more than 5 thousand cancer specimens from 10 different cancer types. Our results show that, in many cases, snoRNA and host gene-genetic alterations are coupled, but exceptions exist and should be considered when analyzing this kind of data.

## 2. Materials and Methods

### 2.1. TCGA Data

The data analyzed in this study were a TCGA (The Cancer Genome Atlas) project dataset of 5359 patient-derived tumors representing 10 distinct human cancers (Table 1). For all of our TCGA study cases we analyzed the copy number counts and mRNA expression data, obtained from the cBioportal for Cancer Genomics (http://www.cbioportal.org/) [23,24]. The cBioPortal provides multidimensional cancer genomics data. The 10 cancer cohorts were chosen based on sample size and the availability of data regarding each pair of host-gene and snoRNA. In Table 1, the TCGA abbreviations for each tumor type with total sample number per tumor type are reported.

The copy number alteration (CNA) dataset, downloaded from cBioPortal, was preprocessed using GISTIC2.0 [25]. The GISTIC pipeline allows to identify sections of the genome that are significantly amplified or deleted across a set of samples. The expression levels were quantified by RSEM (RNA-Seq by Expectation Maximization) [26], an accurate tool for quantifying transcript abundances from RNA-seq data; also these data were obtained from cBioPortal.

We calculated the overall percentage of samples with copy number alterations co-occurring in host genes and snoRNA that belonged to 295 pairs extracted from the snoDB database (http://scottgroup.med.usherbrooke.ca/snoDB/) [27]. This provided us with alteration frequencies for each pair of host gene and snoRNA in ten human cancer types (Supplementary Table S1).

### 2.2. Copy Number Alterations and Gene Expression Data

The copy number status by sample was reported as −2 (Deep Deletion), −1 (Shallow Deletion), 0 (Diploid status), 1 (Gain), 2 (Amplification) levels. Deep deletions refer to the homozygous deletions; Shallow deletion indicates a shallow loss e.g., heterozygous deletion; Diploid is the normal status; Gains refers to a low-level copy gain of gene; Amplifications indicate a high-level gene amplification. Gene expression levels were quantified by RSEM from RNA-Seq data and mRNA Z scores were computed using the tumors samples that were diploid for the corresponding gene. For each gene, Z score is equal to the difference between the specific expression of this gene in a specific sample and the average expression, over the standard deviation of this gene across samples, respectively. We classified as upregulated those genes with Z scores greater than 2 and downregulated those genes, with Z scores less than −2 (Supplementary Table S2).

### 2.3. Statistical Analyses

Statistical analysis was performed using R (version 3.6.1) including R packages of data.table for data cleaning and management, tidyr for data clean-up, and ggplot2 for data visualization. Fisher’s exact test was used to assess associations between categorical variables (significant with *p*  <  0.05).

## 3. Results

### 3.1. snoRNAs and snoRNA Host Genes Alterations Vary Significantly in Different Cancer Types

We first compiled a sno-RNA/host gene shortlist starting from the snoDB public database (http://scottgroup.med.usherbrooke.ca/snoDB/) [27]. A total of 295 snoRNA-host gene couples were chosen on the basis of the following criteria: 1. The snoRNA was included in the intronic region of either a protein-coding or a long non-coding mRNA gene; 2. the ensemble ID was available for the host gene and for the snoRNA. For the genes present in the shortlist, we analyzed the mutational data available from The Cancer Genome Atlas (TCGA) data portal relative to ten different cancer types: breast invasive carcinoma (BRCA), colorectal adenocarcinoma (COAD), glioblastoma multiforme (GBM), head and neck squamous cell carcinoma (HNSC), lung adenocarcinoma (LUAD), lung squamous cell carcinoma (LUSC), ovarian serous cystadenocarcinoma (OV), uterine corpus endometrial carcinoma (UCEC), skin cutaneous melanoma (SKCM), and kidney renal clear cell carcinoma (KIRC) (Table 1). These cancer types were specifically as they were found to contain higher mutation rates for the targets of interest.

**Figure 1:**
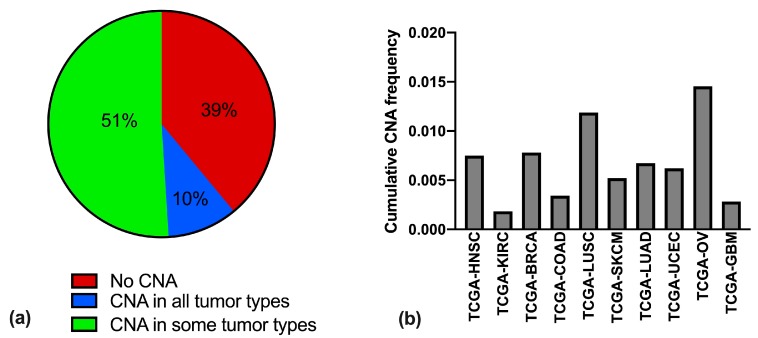
Occurrence of snoRNA/host gene alterations in the ten groups of cancers. (**a**) Percentage of copy number alterations (CNA) in snoRNA/host gene couples, across ten different tumor types (breast invasive carcinoma (BRCA), colorectal adenocarcinoma (COAD), glioblastoma multiforme (GBM), head and neck squamous cell carcinoma (HNSC), lung adenocarcinoma (LUAD), lung squamous cell carcinoma (LUSC), ovarian serous cystadenocarcinoma (OV), uterine corpus endometrial carcinoma (UCEC), skin cutaneous melanoma (SKCM), and kidney renal clear cell carcinoma (KIRC)) divided between couples never undergoing CNA (red), undergoing CNA in all tumors (blue), and undergoing CNA only in some tumor types (green). (**b**) Cumulative CNA frequency in the ten tumor types, calculated in each series as total number of CNA events divided for the total number of possible alterations (i.e., the number of snoRNA/host gene couples (295) times the number of patients in the series).

We analyzed the cumulative copy number alteration (CNA) frequency for each snoRNA/host gene couple, for the different tumor types, in terms of high-level amplifications or homozygous deep deletions. For 39% of the couples no CNA was detected in any of the tumor types queried (Table S1), suggesting that CNAs in each of these genes might confer a disadvantage in cell growth; for 10% of the couples CNA was detected to some extent throughout all of the different tumor types, while for the remaining 51% CNA was found in some tumors and not in others (Figure 1a).

The tumor types with the higher snoRNA/host gene CNA frequencies turned out to be those of the OV group, followed by LUSC, BRCA, HNSC, and LUAD. The tumor types with the lowest cumulative CNA frequency were KIRC and GBM (Figure 1b).

### 3.2. Copy Number Alterations of snoRNAs and Matched Host Genes are Not Always Coupled

**Figure 2:**
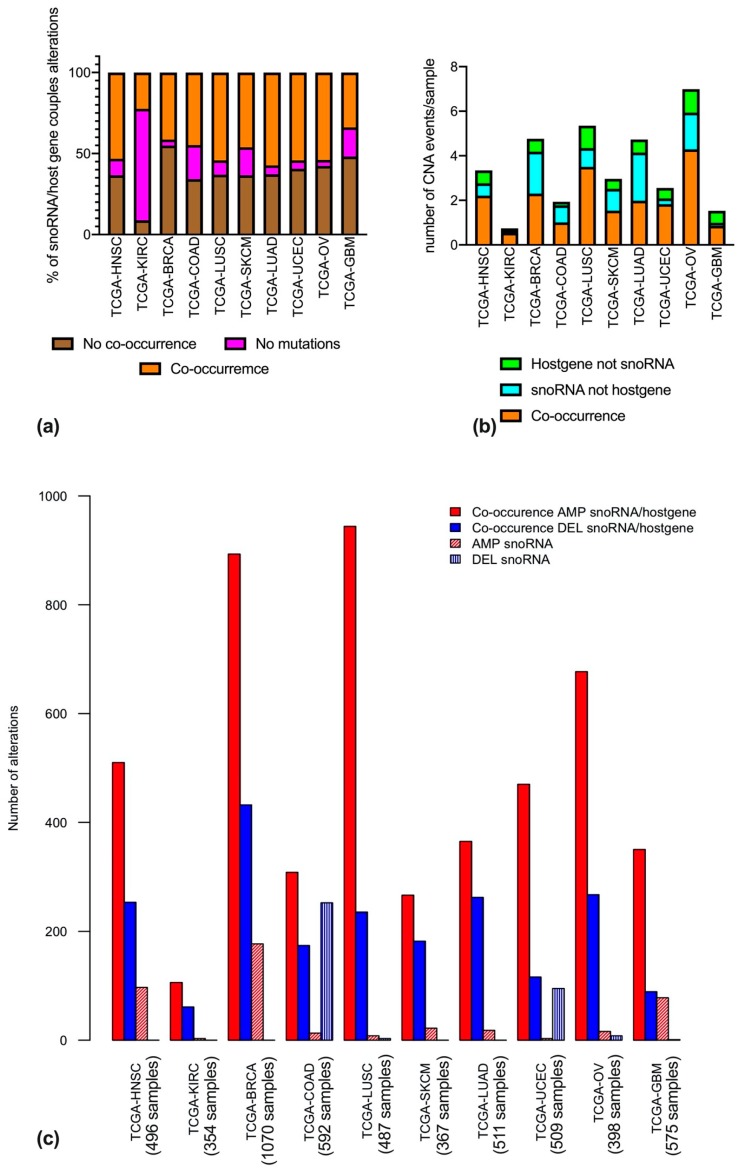
Co-occurrence of CNA in snoRNAs and host genes across different cancer types. (**a**) Percentage of co-occurrence of CNA in snoRNAs and host genes. Orange bars represent co-occurring mutations; brown bars represent mutations that do not co-occur, and purple bars represent absence of mutations. (**b**) Number of CNA events, normalized per sample, and represented with different colors in case of co-occurrence (orange), alteration in snoRNA but not in the host gene (light blue) or alteration in the host gene but not in the snoRNA (green). (**c**) Number of mutational events, split into amplification (red) and deletion (blue), and further into CNAs only involving snoRNAs (textured bars) and CNAs involving both snoRNA and host gene (filled bars).

As the snoRNAs considered herein map within introns of other genes, it would be reasonable to postulate a concordance in their copy number alterations. However, the data we analyzed show that, in many cases, these assumptions are not correct. Indeed, when analyzing the CNA data for the snoRNA/host gene couples separately for each tumor type, it is clear that a co-occurrence of an amplification or deletion is not always the most frequent event. Indeed, there are cases where an amplification or deletion of the snoRNA does not co-occur with the same event in the host gene, and vice-versa (Figure 2a). Co-occurrence of CNA happened for about half of the mutant couples in the majority of the tumors, with striking exceptions for KIRCs (where co-occurrence happens 3 times more frequently) and BRCAs (where in more than half of the couples, alterations are not co-occurring).

In addition, we wondered whether, when taking into consideration those alterations that fail to co-occur, these would be more frequently related to the snoRNA or to the host gene. To answer this question, we analyzed the average number of alterations per tumor sample. Strikingly, this analysis revealed that the average number of alterations per sample varied greatly among different tumor types, with the lowest number in KIRCs, and the highest in OVs (Figure 2b). Secondly, CNAs in snoRNA not occurring in host genes were more frequent in BRCAs, OVs, LUADs, COADs, and SKCM, whereas CNAs in host genes not co-occurring in snoRNAs were more frequent in GBMs, UCECs, and LUSCs. In KIRCs and HNSCs the two events were found to be equally frequent (Figure 2b).

We next characterized the CNA events by identifying them as either amplifications or deletions. Within each tumor type, the number of amplifications or deletions in the snoRNA only group, and in the co-occurrence group was summed. Figure 2c represents the total number of each type of alteration, and is, by definition, influenced by the multiplicity of the series for each tumor type.

### 3.3. Frequently Altered snoRNA-Host Gene Couples Recur in Different Cancer Types

Apart from these general considerations, it is of particular interest to identify those snoRNA/host gene couples which vary more frequently across different tumor types. Indeed, among these there may be interesting targets with diagnostic or prognostic potential or useful for risk stratification of patients.

**Figure 3:**
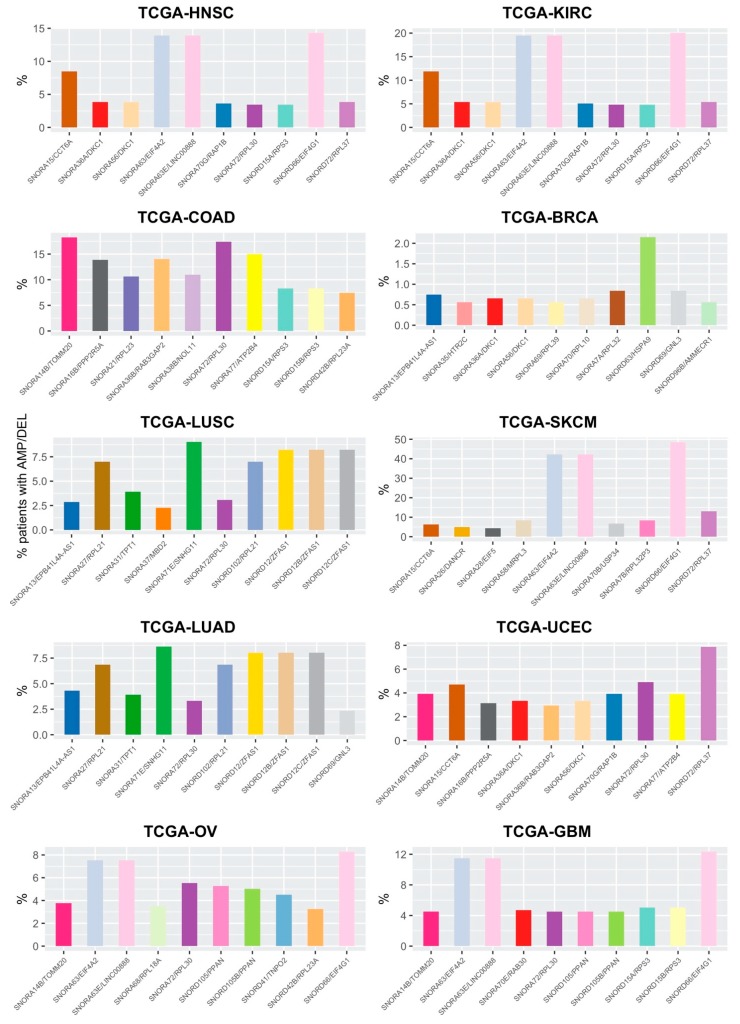
Top snoRNA/host gene couples undergoing CNA in the ten different tumor types. For ease of interpretation, couples recurring in different tumor types have been assigned the same bar colors.

For each tumor type, we selected a shortlist of the 10 snoRNA/host gene couples which undergo CNA most frequently in patients. As shown in Figure 3, some snoRNA/host gene couples recur in the top ten of more frequently altered couples, albeit with different frequencies, in different cancer types. The most prominent examples are those of SNORA63/EIF4A2, SNORA63E/LINC00888, SNORD66/EIF4G1. These couples undergo CNA in a significant portion of patients in HNSC, KIRC, SKCM, OV, and GBM; in all these tumor types, these 3 couples are altered in similar percentages of patients, ranging from 7–8% in OV cancer, to over 40% in SKCM. Other couples recurring in multiple cancer types are SNORA15/CCT6A, altered in 5–12% of cases of HNSC, KIRC, SKCM, and UCEC; SNORA14B/TOMM20, altered in close to 20% of COADs and, to a lower extent, in OV, UCEC, and GMB; SNORD72/RPL37, altered in over 10% of SKCM and, to a lower extent (4–8%) in HNSC, KIRC, and UCEC; SNORA56 and SNORA36A/DKC1, mutated in 0.5–5% of HNSC, KIRC, BRCA, and UCEC patients; and SNORA70G/RAP1B, altered in 4–5% of patients in HNSC, KIRC, and UCEC.

Another aspect worthy of consideration is the very high overlap of genes mutated in the two lung cancer types assayed, adenocarcinoma and squamous cells carcinoma. In the top 10 altered couples for these tumor types, 9 are identical: SNORA13/EBP41L4A-AS1, SNORA27/RPL21, SNORA31/TPT1, SNORA71E/SNHG11, SNORA72/RPL30, SNORD102/RPL21, SNORD12/ZFAS1, SNORD12B/ZFAS1, and SNORD12C/ZFAS1. Strikingly, alteration frequencies of the 9 overlapping couples in the two tumor types are extremely similar, and range between 2.5% and 10% of patients.

It is also interesting to note that BRCA, which is in the group of those with a higher recurrence of CNA per patient (Figure 2b), does not have any snoRNA/host gene couple recurring in more than 2.5% of the patients (Figure 3). This may indicate that, in this specific pathology, alterations in snoRNAs and host genes may be related to a generalized genomic instability, and may therefore play a role as passenger mutations.

Other snoRNA/host gene couples identified in the ten shortlists of frequently altered targets are shown in Figure 3 and are not detailed in the text.

### 3.4. Differential ExpressionAalterations of snoRNAs and Host Genes in Multiple Cancer Types

**Figure 4:**
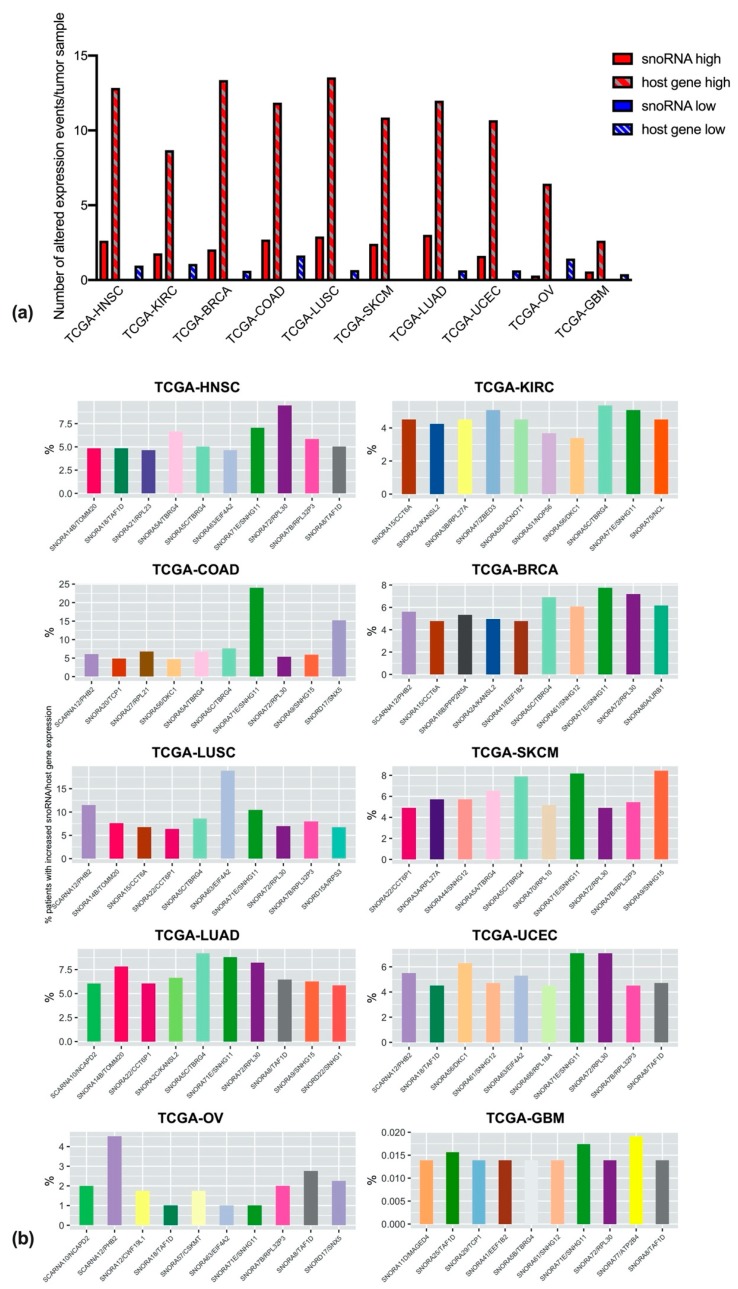
Alterations in gene expression for snoRNAs and host genes. (**a**) Number of expression alteration events, normalized per sample, and represented with different colors in case of higher expression (red), lower expression (blue), and further split into expression alterations only involving snoRNAs (filled bars) or involving host genes (textured bars). (**b**) Top snoRNA/host gene couples undergoing expression alterations in the ten different tumor types. For easier interpretation, couples recurring in different tumor types have been assigned the same bar colors, and colors are matched to those assigned in Figure 3.

We reasoned that copy number alterations likely impacts upon the differential expression of snoRNAs and host genes. Therefore, we extracted expression data for the targets of our interest from TCGA datasets. Data were analyzed in order to discriminate between over- or under-expression of snoRNAs and host genes; over- or under-expression was determined by comparing the expression levels of each gene in the cancer population to those of the same gene in the reference population (constituted by all tumor samples profiled for that specific gene in TCGA). Figure 4a shows the average number per sample of over- or under-expression events, those events involving the host gene or the snoRNA being considered separately. As is clear from the image, over-expression events are much more frequent when compared to under-expression, and in general host-gene over-expression is detected 4–6 times more than snoRNA over-expression (with the exception of OV cancers, where the frequency for host gene over-expression is 20 times higher). In addition, it is striking that snoRNA-down regulation is never detected.

We next looked at the cumulative frequency of expression alterations for the snoRNA/host gene couples. Since transcriptional down-regulation turned out to be a relatively infrequent event for our targets (Figure 4a), we decided to further analyze only the up-regulated snoRNA/host gene couples. Figure 4b shows, for each cancer type, the 10 couples with higher expression alteration frequencies. The first, remarkable observation is that alteration frequencies are fairly constant across different tumor types (and range around 5% for most targets), with the exceptions of GBM, where alteration frequencies are extremely low (below 0.1%), and LUSC and COAD, where, conversely, alteration frequencies are generally higher, arriving to 18–25% for selected targets.

Secondly, as observed for CNAs, some of the targets in the short-lists are common to different tumor types. For instance, SNORA71E/SNHG11 is in the top 10 of over-expressed targets in all tumor types, SNORA72/RPL30 is in the top 10 for all tumor types but KIRCs and OVs, and SNORA5C/TBRG4 is only missing in UCECs, OVs, and GBMs shortlists.

Furthermore, it is worthwhile to note that less than half of the over-expressed targets are present in the shortlist of the couples undergoing CNA, suggesting that, in many cases, large amplifications and deletions of snoRNAs and of their host genes do not directly impact upon their expression.

## 4. Discussion

Somatic DNA copy number alterations (CNA) are nearly ubiquitous in cancer [28,29] and alter a greater portion of the cancer genome than any other type of somatic genetic alteration, playing important roles in oncogenesis and cancer therapy [29,30]. CNA can influence cancer gene expression regulation in several ways. There is indeed evidence that increased copy number can positively or negatively impact upon transcription, by altering dosage or by disrupting proximal or distant regulatory regions [31,32].

A growing number of reports in the literature have recognized a close connection between snoRNA alterations and cancer; however, many snoRNA genes map to intronic regions of other genes, and in most (but not all) cases, alterations occurring in snoRNAs are closely related to alterations in their host genes. Therefore, we believe that an analysis of the effects of snoRNAs alterations should always take into account the possible, co-occurring alteration of the matched host gene. This consideration is at the basis of the study presented here.

Indeed, several snoRNA-containing host genes are acknowledged players in cancer development. For instance ribosomal proteins (RPs), like RPL5, which has been shown to be a tumor suppressor frequently deleted or altered in multiple cancer types, (reviewed in [33], or of other RPs, [34]. Likewise, translation initiation (EIF1A, EIF4A, EIF4G) and elongation (EEF1B2, EEF2) factors are well-known to play a role in cancer [35,36], as well as ribosome biogenesis related factors, like the pseudouridine synthase dyskerin [37] and the 2′-*O*-methyltransferase fibrillarin [38]. In addition, non-coding host genes have recently been found to be involved in cancer onset or progression, for example ZFAS1 [39], GAS5 [40], and MEG8 [41]. On top of these, multiple host genes are known regulators of cell metabolism, cell cycle, cell adhesion, and cell signaling, and their de-regulation therefore may be involved in cancer development.

When considering CNA, amplification of snoRNA and host genes sequences were found to be most frequent event, with deep deletions occurring less frequently, suggesting that, in the majority of cases, in order to sustain their growth cancer cells, may take advantage of snoRNA/host genes overexpression. This finding is in contrast with a previous study, performed on TCGA datasets, which indicated that CNA deletions, and not amplifications, were the more frequent event, [42], but our observations fit well with the known functions of these genes in support of cell growth [3].

In our analysis, a relevant proportion (almost 40%) of snoRNA/host gene couples never turned out to be altered in any tumor type, suggesting that these genes are essential for basic cellular functions conserved within cancer cells, or, alternatively, that they do not play any significant role in neoplastic transformation. Conversely, 10% of snoRNA/host genes couples are found altered in all the tumor types considered (although with important frequency variability amongst different tumors), suggesting that their dysregulation might contribute to tumor development independently of the tissue of origin.

Intriguingly, we found surprising the result that some CNA events do not co-occur in intronic snoRNA and matched host gene. This event was found more frequently in the sense of CNA of the host gene as opposed to the snoRNA, but in some cases also in the converse direction. This unexpected finding could perhaps be explained by the postulation that snoRNAs are mobile genetic elements and therefore in tumors they may duplicate or insert in different positions of the genome [43].

Our analyses highlighted different CNA events, involving intronic snoRNAs and matched host genes, which are worth consideration. Among these, we found an overlap in the amplification frequencies of three different snoRNA/host gene couples, namely SNORA63/EIF4A2, SNORA63E/LINC00888, SNORD66/EIF4G1, in five different cancer types: HNSC, KIRC, SKCM, OV, and GBM. All these genes map to the same chromosomal location, 3q27, which also hosts different oncogenes (like TERT, PI3KCA, and BCL6) and has been shown to be frequently amplified in squamous cell carcinomas with different localizations (lung [44], esophagus [45], mouth [46]), in lymphomas [47], and in lung cancers different from LUSC (i.e., small cell lung carcinomas and adenocarcinomas [48]). Although it is difficult to speculate a possible mechanism for specific snoRNA/host gene CNA contribution to cancer onset, it is worthwhile to consider that the snoRNAs/host gene couples highlighted for being altered in multiple cancer types (Section 3.3) have already been recognized for having a specific biological relevance, which could be linked to clinical relevance in many cancer types (Table 2).

When considering expression data for the three overlapping snoRNA/host gene couples mentioned above, only the SNORA63/EIF4A2 turned out to be over-expressed in squamous cell carcinomas of head and neck and of the lung, suggesting either that in most cancer types snoRNA/host gene CNA is an early event in tumor development, and expression of the two is first boosted, and later on is shut down, or alternatively that amplification of SNORA63/EIF4A2 is a passenger event, consequent to the proximity to other cancer driver genes in the chromosome. Even though further studies are necessary to shed light on this matter, a role for EIF4A2 in lung cancer and in other cancer types has been previously proposed [50,74,75], indicating that these mutations might have a driver, rather than passenger, role in tumorigenesis. In addition, in the datasets we analyzed, the overexpression of snoRNA63 is detectable in half of the EIF4A2 overexpressing LUSC patients and in about one fourth of the HNSC patients, suggesting that co-expression of the host gene and the intronic snoRNA is not co-regulated, and implying a possible, still unexplored, role for SNORA63 overexpression in cancer. Similar considerations can of course be extended to other snoRNA/host gene couples, for which a correspondence in CNA and expression is not apparent. Among these, SNORA15/CCT6A, which we found genetically altered in a high percentage of HNSC, KIRC, SKCM and UCEC patients, but overexpressed in around 5% of SKCM, BRCA, and LUSC patients (with an amplification/overexpression correspondence only for melanomas, where CCT6A overexpression has previously been linked to drug resistance [76], but the contribution of SNORA15 remains unexplored).

In addition, our expression analysis identified two snoRNA/host gene couples (SNORA71E/SNHG11 and SNORA5C/TBRG4) which are transversally up-regulated in different cancer series, indicating that they may have a role in cellular mechanisms of tumorigenesis. Even though the relevance of these two snoRNAs has not yet been reported, different groups have reported alterations in SNHG11 and TBRG4 in different cancer types [77,78,79,80,81].

One unexpected result that we observed in tumors is that downregulation has never been observed for any snoRNA within the queried datasets. This is particularly surprising when taking into account that, in a limited number of cases, snoRNA sequences were found to be subject to deep deletions. Importantly, the majority of TCGA-based studies analyzed snoRNA expression from library preparation methodologies that enrich for small RNAs (less than 200 nucleotides in length), implying the loss of a large fraction of snoRNAs of middle-to-large size [19]. The frequency of these alterations could therefore be largely underestimated and perhaps some of the surprising results observed in snoRNA expression datasets (including those of over-expression) ought to be reconsidered in light of these methodological limitations.

A previous large study interrogated the TCGA database for snoRNA CNA, expression alteration and gene methylation on more than 10,000 samples across 31 cancer types [19]. This study identified 46 snoRNAs relevant for human cancer. Importantly, 9 of these snoRNAs were also identified by our analysis with a different methodological approach, namely SNORA21, SNORA56, SNORD12B, SNORD12C, SNORD41, SNORD15A, SNORD15B, SNORD72, SNORD102, confirming the observation that these snoRNAs play an important role in cancer development. However, with the exception of the few selected cases discussed above, for most of the observed alterations precise mechanistic insights regarding their role in tumorigenesis are currently lacking. In principle, changes in the expression of guide snoRNAs should impact upon the modification of their specific target site. The majority of cases involve rRNA, consequently ribosomal activity may be affected, potentially impacting on the translatome. However snoRNAs are known to have different activities such as regulation of splicing and mRNA abundance tRNA methylation, etc. (see [15] for review), therefore changes in their expression may modify gene expression at multiple levels.

To understand how alterations of snoRNA genes can contribute to cancer, new studies implementing adequate technical approaches on tumor material of human origin are required to allow for the characterization of the biological significance of mutation and/or alteration of the expression of snoRNAs and their host genes.

## 5. Conclusions

Altogether, our findings suggest that studies analyzing CNA and/or expression deregulation of snoRNA genes and/or of host genes, should take into account the differential contribution to cancer development of both snoRNAs and host genes. Indeed, snoRNAs and their host genes may contribute to cancer onset, progression, and response to therapies in conjunction or independently.

## Figures and Tables

**Table 1:** Summary of the tumor types, with acronyms and numerosity.

Tumor Acronym	Tumor Type	Sample Number
TCGA-HNSC	Head and Neck squamous cell carcinoma	496
TCGA-KIRC	Kidney renal clear cell carcinoma	354
TCGA-BRCA	Breast invasive carcinoma	1070
TCGA-COAD	Colorectal adenocarcinoma	592
TCGA-LUSC	Lung squamous cell carcinoma	487
TCGA-SKCM	Skin Cutaneous Melanoma	367
TCGA-LUAD	Lung adenocarcinoma	511
TCGA-UCEC	Uterine Corpus Endometrial Carcinoma	509
TCGA-OV	Ovarian serous cystadenocarcinoma	398
TCGA-GBM	Glioblastoma multiforme	575

**Table 2:** Relevance of snoRNA/host gene couples for which CNAs recur more frequently in multiple cancer types.

snoRNA Host Gene	Known Target	Biological Relevance	Clinical Relevance	References
SNORA63 EIF4A2	28S rRNA U4390 (Helix 89) --	Control of translation initiation, elongation and termination Translation initiation	None reported Inversely correlates with prognosis in COAD, NSCLC, BRCA	[49,50,51]
SNORA63E LINC00888	Unknown --	--	None reported Increased in SKCM	[52]
SNORD66 EIF4G1	18S rRNA C1272 (Helix 32) --	Translation initiation Translation initiation	Biomarker of NSCLC in sputum, plasma and tissue; correlates to overall survival in NSCLC Inversely correlates with prognosis in BRCA, OV, NSCLC, PDA	[53,54,55,56,57,58]
SNORA15 CCT6A	18S rRNA U1367 (Helix 37) --	Activation of TGFbeta signaling	None reported Correlates with negative prognosis in HCC	[59,60]
SNORA14B TOMM20	18S rRNA U966 (Helix 23) --	E site/translation elongation Increases mitochondrial ATP synthesis	None reported Overexpressed in various cancer types; potential therapeutic target in COAD	[61,62]
SNORD72 RPL37	5.8S rRNA U55 (Helix 6) --	Formation of the ribosome small subunit Activator of ribosomal stress pathway	None reported Overexpressed in PC	[33,63,64]
SNORA56 DKC1	28S rRNA U1664 (Helix 37) --	Putative mRNA/tRNA binding rRNA pseudouridylation; regulates translational fidelity and Cap-independent translation; Telomere binding	None reported Negatively correlates with survival in BRCA, and NSCLC	[37,65,66,67,68]
SNORA36A DKC1	18S rRNA U105 (Helix 7) and U1244 (Helix 31) --	Binding of factors to form the 90S preribosome (H7); Translation elongation (H31) See above	None reported See above	[69,70]
SNORA70G RAP1B	18S rRNA U1692 (Helix 28) --	Small ribosomal subunit maturation; translation initiation GTPase regulating cell adhesion, migration, polarity, differentiation, growth and angiogenesis	None reported Negatively correlates with prognosis in GC; up-regulated in ESCC	[71,72,73]

COAD: colon adenocarcinoma; NSCLC: non-small cell lung carcinoma; BRCA: breast cancer; SKCM: skin cutaneous melanoma; OV: ovarian cancer; PDA: pancreatic ductal adenocarcinoma; HCC: hepatocellular carcinoma; PC: prostate cancer; GC: gastric cancer; ESCC: esophageal squamous cell carcinoma.

## Supplementary Materials

The following are available online at https://www.mdpi.com/2073-4409/9/2/387/s1 Table S1: alteration frequencies of CNA, Table S2: snoRNA/host gene expression.
